# Uterine Cavity Abnormalities in Patients with Endometriosis in Alexandria: A Diagnostic Test Accuracy Study

**DOI:** 10.1155/2017/5869028

**Published:** 2017-05-30

**Authors:** Eman Aly Abd El Fattah

**Affiliations:** Department of Obstetrics and Gynecology, Faculty of Medicine, Shatby Maternity Hospital, Alexandria University, Alexandria, Egypt

## Abstract

Endometriosis is strongly associated with infertility. Endometrial polyps are prevalent in infertile women and they have similar pathological characteristics to endometriosis, suggesting a possible association. Uterine malformations as uterine septum and hypoplastic uterus are also linked to endometriosis. Hysterosalpingogram and transvaginal ultrasonography are used to diagnose endometrial lesions. Hysteroscopy can detect small lesions that might be missed. Recently, 4D ultrasonography is being used, but which is superior has not been established yet. We aim to compare 4D ultrasonography to office hysteroscopy in evaluating uterine cavity in cases with endometriosis; also we aim at correlating these findings with the stage of endometriosis. 50 cases of endometriosis diagnosed by laparoscopy were randomly selected from El Shatby fertility clinic, Alexandria University, Egypt, with exclusion of cases with any previous intrauterine surgery or any hormonal treatment. Transvaginal 4D ultrasonography and office hysteroscopy were done. 4D ultrasonography agreed with office hysteroscopy in diagnosing abnormal uterine findings in 14 cases and four additional cases were diagnosed by hysteroscopy alone.* Conclusion*. Endometrial polyps, septate uterus, and hypoplastic uterus are more prevalent among infertile women who happen to have endometriosis. 4D ultrasonography and office hysteroscopy are equally successful in assessing the uterine cavity.

## 1. Introduction

It is estimated that endometriosis occurs in 10% of women during the reproductive years [1]. It has been classified into superficial, deep, and ovarian types. The superficial type may be presented as classical implants and vesicular, popular, nodular, haemorrhagic, healed, and apparently normal peritoneum [2]. It commonly presents between 25 and 29 years of age [1]. It is strongly associated with infertility, which is attributed to distorted adnexal anatomy [3], interference with oocyte development or early embryogenesis [4], or reduced endometrial receptivity [5]. Several studies have suggested impairment of implantation which may be due to intrinsic deficiencies within the uterus [6] and structural or ultrastructural defects [7]. Endometrial polyps are common gynecological disorder whose prevalence is increased in infertile women [8]. The exact pathogenesis of these polyps is not yet known, but the similar pathological characteristics to endometriosis suggest a possible association [9]. Anatomical uterine malformations are also linked to endometriosis [10]. Uterine septum, the most common Müllerian duct anomaly, results in colicky uterine peristalsis and increased menstrual regurgitation through the fallopian tubes [11]. Hypoplastic uterus, a rare anomaly, may be also associated [12]. Both hysterosalpingogram and transvaginal ultrasonography are used to diagnose endometrial lesions but sometimes they are not enough [13]. Hysteroscopy, the gold standard for evaluation of uterine causes of infertility, can detect small lesions that might not otherwise be detected by other methods [14]. In the recent years, office hysteroscopy has been preferred to operative hysteroscopy in routine evaluation of the endometrial cavity [15]. Recently, 4D ultrasonography is being used for the same purpose but which is superior has not been established yet. In this study, we aim to compare between 4D ultrasonography and office hysteroscopy in evaluating uterine cavity in cases with endometriosis.

## 2. Patients and Methods

A prospective observational study was conducted on 50 cases of endometriosis which is the number needed to estimate the prevalence of uterine cavity abnormalities which is equal to 69% [16] with 12% precision (due to high variability at the estimated rates) using an alpha error of 5% [17]. The sample was selected randomly using the simple random technique.

All patients were recruited from El Shatby fertility clinic, Alexandria University. Endometriosis was diagnosed by laparoscopy (discrete endometriotic lesions, endometriomas, or adhesions) [18] or transvaginal ultrasonography for endometriomas (cystic lesion with low level internal echoes, occasional thick septations, thickened walls, and echogenic wall foci) [19]. Patients with history of any previous intrauterine cavity or those receiving any hormonal treatment for the previous six months were excluded.

### 2.1. Methods

Informed consent was signed by all participants; complete history was taken (age, gravidity, parity, menstrual history, and previous surgery; complete examination (general, abdominal, and vaginal) was done. Transvaginal 4D ultrasonography was done in the dorsal lithotomy position. Uterus was assessed in the transverse and sagittal planes, adnexa were scanned for cysts or endometriotic nodules, and tubes were scanned for hydrosalpinx.

Office hysteroscopy was done immediately after menstruation using normal saline as distention media and 5 mm continuous flow sheath with an operative channel for the use of scissors, grasping forceps, or biopsy forceps when necessary. Light was provided by normal cold light source. During the procedure, the patient was placed in the dorsal lithotomy position, vulva and vagina were cleaned with antiseptic solution, the technique avoided the use of a speculum or a tenaculum, the vagina as a cavity was distended using the hysteroscope introduced into its lower part, and the anatomy was followed up till the cervical canal onto the uterine cavity. The uterine cavity was inspected symmetrically and the tubal ostia were identified; then the hysteroscope was pulled backwards to obtain a panoramic view of the whole cavity, and cervical canal was inspected during withdrawal. Findings were recorded. Polyps, when seen, were biopsied with biopsy forceps introduced through the operative channel and the diagnosis was confirmed with histopathological examination.

#### 2.1.1. Statistical Methodology

Data were collected and entered to the computer using SPSS (Statistical Package for Social Sciences) program for statistical analysis (ver. 21) [20]. Data were entered as numerical or categorical, as appropriate. When Kolmogorov-Smirnov test revealed no significance in the distribution of variables, parametric statistics were carried out, while in the not-normally distributed data the nonparametric statistics were carried out [21]:

- Data were described using minimum, maximum, mean, and standard deviation for the normally distributed data.
- Interrater (interobserver) agreement was carried out using kappa and weighted kappa tests [20, 22]. Proportions of specific agreement were calculated according to Spitzer and Fleiss [23].

An alpha level was set to 5% with a significance level of 95%, and a beta error was accepted up to 20% with a power of study of 80%.

#### 2.1.2. Agreement Analysis

Magnitude guidelines have appeared in the literature. Perhaps the first were Landis and Koch [24] who characterized values < 0 as indicating no agreement and 0–0.20 as slight, 0.21–0.40 as fair, 0.41–0.60 as moderate, 0.61–0.80 as substantial, and 0.81–1 as almost perfect agreement. This set of guidelines is, however, by no means universally accepted; Landis and Koch supplied no evidence to support it, basing it instead on personal opinion. It has been noted that these guidelines may be more harmful than helpful [25]. Fleiss's [26] equally arbitrary guidelines characterize kappa over 0.75 as excellent, 0.40 to 0.75 as fair to good, and below 0.40 as poor.

## 3. Results

**Figure 1:**
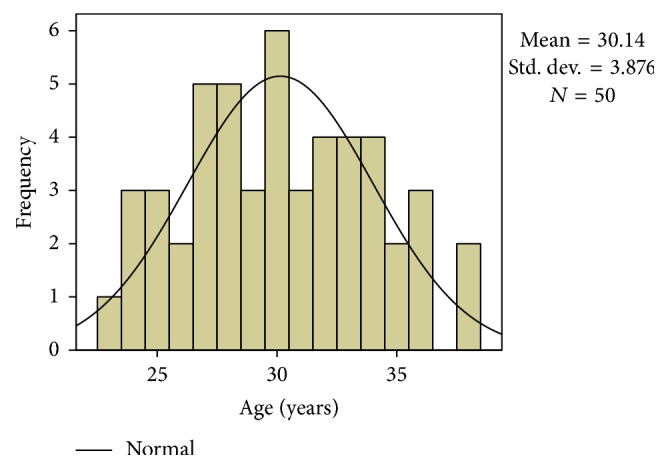
Histogram showing age distribution.

The study was conducted upon fifty cases of endometriosis; their age ranged from 23 to 38 years of age with a mean of 30 ± 3.876 years (Figure 1). 42 cases complained of primary infertility, while 8 cases complained of secondary infertility. By laparoscopy, we had 8 cases in stage I, 17 cases in stage II, 4 cases in stage III, 20 cases in stage IV, and one missing case. Endometriomas were found in 38 cases only (Table 1).

By 4D ultrasonography, we had abnormal uterine findings in 14 cases and this was confirmed by office hysteroscopy. On the contrary, 36 cases were completely normal by 4D ultrasonography but, by office hysteroscopy, 3 cases had abnormal findings which are statistically significant (Table 2). 4D ultrasonography had sensitivity of 82.35% and specificity of 100% for diagnosing abnormal uterine findings with a positive predictive value of 100% and a negative predictive value of 91.67% and an overall test accuracy of 94%.

Abnormal findings were in the form of polyps, septate uterus, and hypoplastic uterus.* Endometrial polyps* were found in 8 cases by 4D ultrasonography which was confirmed by hysteroscopy (positive agreement), and 4D ultrasonography diagnosed 42 cases as being normal which also agreed with hysteroscopy; only 2 cases were positive for the presence of polyps by hysteroscopy and they were diagnosed as free by 4D ultrasonography (Table 3). 4D ultrasonography had sensitivity of 80.00% and specificity of 95.24% for diagnosing endometrial polyps with a positive predictive value of 80% and a negative predictive value of 95.24% and an overall test accuracy of 92.31%.

*Septate uterus* was found in only 4 cases by 4D ultrasonography against 46 free cases. This was proven by hysteroscopy (positive agreement) in addition to one case which was diagnosed as free by 4D ultrasonography, but a septum was found on hysteroscopy (Table 4). 4D ultrasonography had sensitivity of 80.00% and specificity of 100.00% for diagnosing septate uterus with a positive predictive value of 100.00% and a negative predictive value of 97.83% and an overall test accuracy of 98.00%.

*Hypoplastic uterus* was found in only 2 cases by 4D ultrasonography against 48 free cases which was the same result obtained by hysteroscopy (positive agreement) (Table 5). 4D ultrasonography had sensitivity of 100.00% and specificity of 100.00% for diagnosing hypoplastic uterus with a positive predictive value of 100.00% and a negative predictive value of 100.00% and an overall test accuracy of 100.00%.

## 4. Discussion

Endometriosis is a common gynecological disorder; its association with infertility is complex and controversial. Virtually, every aspect of reproduction in women with endometriosis has been investigated. Although advanced stages may manifest easily recognizable infertility factors, such as tubal distortion or obstruction, the mechanisms underlying reproductive dysfunction in women with minimal or mild disease are more subtle [27]. Many studies were conducted on endometriosis patients and many hypotheses exist to explain the relation between it and infertility but still the precise mechanism remains unclear. Our study aimed at evaluating the uterine cavity in those patients by 4D ultrasonography and by hysteroscopy and we also aimed at comparing these two methods to each other. 4D ultrasonography has the advantage of exact volume measurement of endometrial polyps which cannot be achieved by 2D ultrasonography. We succeeded in proving that 4D ultrasonography could be a good diagnostic tool for diagnosing abnormal uterine findings with a positive predictive value of 100% and an overall test accuracy of 94%. Hypoplastic uterus was the main abnormal uterine finding that could be detected with 4D ultrasonography with an overall test accuracy of 100% but unfortunately this was not the case for endometrial polyps where the overall test accuracy was 92.31%

After searching the literature, we found two studies reporting high prevalence of endometrial polyps in endometriosis patients [16, 28] and one study reporting uterine anomalies [29]. These studies aimed at just finding a relation or an association between abnormal uterine cavity findings and endometriosis and they did not recommend a particular diagnostic tool but, on the other hand, they used 2D ultrasonography, hysterosalpingography, and hysteroscopy without showing which of these methods was superior to the others.

Functional endometrial polyps which are confined to hormonally responsive layer of the uterus are estrogen-dependent. The exact pathogenesis of these benign lesions is not well known, but hormonal dysfunction is the most accepted theory [30]. In a study done by Hinckley and Milki upon 1000 infertile patients in 2004, prevalence of endometrial polyps was 35% only [29]. A larger study conducted upon 2500 infertile patients in 2010 reported prevalence of 7.86% [8]. In this study, researchers recommended hysteroscopy in comparison to ultrasonography for diagnosing endometrial polyps but they used the 2D ultrasonography and not the 4D one. The similar characteristics of endometriosis and endometrial polyps suggest a possible association between them. In 2003, Kim et al. studied one hundred eighty-three infertile women: 92 of them had endometriosis and 91 were control. They found polyps in 43 women in the endometriotic group (46.7%) against 15 women in the control group (16.5%) [28].

In 2011, Shen et al. studied 431 cases: 158 cases with endometriosis and 273 cases without endometriosis. Endometrial polyps were found in 108/158 cases (68.35%) with endometriosis and in 56/273 cases of the control group (20.51%) [16]. These results agree with our results as regards the high prevalence of polyps in endometriosis patients. The difference in the prevalence could be due to the number of characteristics and the different population, as the studies were held in China with the consideration of the high prevalence of endometrial polyps in this population.

Our study also evaluated the uterine cavity for uterine anomalies and we reported five cases of septate uterus (10%) and two cases of hypoplastic uterus (4%). The incidence of congenital uterine anomalies in the general population is 0.1–3% [31].

Hinckley and Milki studied 1385 infertile cases, of which 7 cases (0.5%) had uterine septum [29]. Matalliotakis et al. studied 425 cases with endometriosis and 200 cases without endometriosis in 2010. They found 13 cases from 425 having a uterine septum, representing 3%. The control group had only one case, representing 0.5% of the studied group [32]. This agrees with our results, but they did not differentiate between the different diagnostic modalities as we did. Comparing 4D ultrasonography to office hysteroscopy, a positive agreement was found, where only one case of septate uterus was missed by 4D and reported by office hysteroscopy; otherwise both reported the same findings as regards polyps and hypoplastic uterus. This proves that 4D ultrasonography with its advantage as an easy noninvasive technique could be used instead of office hysteroscopy.

## 5. Conclusion

From the present study, we concluded that endometrial polyps, septate uterus, and hypoplastic uterus are more prevalent among infertile women who happen to have endometriosis. Both 4D ultrasonography and office hysteroscopy are equally successful in assessing the uterine cavity but ultrasonography has the advantage of being an easy noninvasive maneuver.

## Figures and Tables

**Table 1:** Frequency of infertility and stage of endometriosis and endometrioma in the studied patients.

	*n*	%
*Infertility*		
(i) Primary	42	84.0
(ii) Secondary	8	16.0
*Stage*		
(i) I	8	16.3
(ii) II	17	34.7
(iii) III	4	8.2
(iv) IV	20	40.8
*Endometriosis*		
(i) Negative	38	76.0
(ii) Positive	12	24.0

**Table 2:** Overall diagnosis (success of hysteroscopy to diagnose polyps as well as uterine anomalies).

	Hysteroscopy		Total
	Negative	Positive	
4D U/S			
Negative	33 (66.0%)	3 (6.0%)	36 (72.0%)
Positive	0 (0.0%)	14 (28.0%)	14 (28.0%)

Total	33 (66.0%)	17 (34.0%)	50 (100.0%)

Kappa	0.860		
Standard error	0.077		
*p* value	0.000^*∗*^		

Weighted kappa	0.860		
Standard error	0.077		
95% CI	0.709–1.000		

*Proportions of specific agreement*: (i) negative agreement = 2*∗*33/(2*∗*33 + 3 + 0) = 95.65%; (ii) positive agreement = 2*∗*14/(2*∗*14 + 3 + 0) = 90.32%. *∗* indicates significance.

**Table 3:** Endometrial polyps.

	Hysteroscopy		Total
	Negative	Positive	
4D U/S			
Negative	40 (80.0%)	2 (4.0%)	42 (84.0%)
Positive	0 (0.0%)	8 (16.0%)	8 (16.0%)

Total	40 (80.0%)	10 (20.0%)	50 (100.0%)

Kappa	0.865		
Standard error	0.093		
*p* value	0.000^*∗*^		

Weighted kappa	0.865		
Standard error	0.093		
95% CI	0.683–1.000		

*Proportions of specific agreement*: (i) negative agreement = 2*∗*40/(2*∗*40 + 2 + 0) = 97.56%; (ii) positive agreement = 2*∗*8/(2*∗*8 + 2 + 0) = 88.89%. *∗* indicates significance.

**Table 4:** Uterine septum.

	Hysteroscopy		Total
	Negative	Positive	
4D U/S			
Negative	45 (90.0%)	1 (2.0%)	46 (92.0%)
Positive	0 (0.0%)	4 (8.0%)	4 (8.0%)

Total	45 (90.0%)	5 (10.0%)	50 (100.0%)

Kappa	0.878		
Standard error	0.120		
*p* value	0.000^*∗*^		

Weighted kappa	0.878		
Standard error	0.120		
95% CI	0.643–1.000		

*Proportions of specific agreement*: (i) negative agreement = 2*∗*45/(2*∗*45 + 1 + 0) = 98.90%; (ii) positive agreement = 2*∗*4/(2*∗*4 + 1 + 0) = 88.89%. *∗* indicates significance.

**Table 5:** Hypoplastic uterus.

	Hysteroscopy		Total
	Negative	Positive	
4D U/S			
Negative	48 (96.0%)	0 (0.0%)	48 (96.0%)
Positive	0 (0.0%)	2 (4.0%)	2 (4.0%)

Total	48 (96.0%)	2 (4.0%)	50 (100.0%)

Kappa	1.000		
Standard error	0.000		
*p* value	0.000^*∗*^		

Weighted kappa	1.000		
Standard error	0.000		
95% CI	1.000-1.000		

*Proportions of specific agreement*: (i) negative agreement = 2*∗*48/(2*∗*48 + 0 + 0) = 100.0%; (ii) positive agreement = 2*∗*2/(2*∗*2 + 0 + 0) = 100.0%. *∗* indicates significance.
